# A Modified “Suspender‐Type” Traction‐Preservation and Native Ligament Reconstruction Using Hamstring Autograft for Anterior Cruciate Ligament Femoral‐Side Avulsion

**DOI:** 10.1002/atn2.70234

**Published:** 2026-08-22

**Authors:** Haiwei Yan, Xiaoling Qin

**Affiliations:** ^1^ Department of Sports Medicine Liuzhou Worker's Hospital/The Fourth Affiliated Hospital of Guangxi Medical University Liuzhou China

## Abstract

Anterior cruciate ligament rupture, a common sports‐related knee injury, often causes instability and functional impairment and increases the long‐term risk of osteoarthritis. Traditional remnant‐preserving anatomical reconstruction is the first‐line treatment for midsubstance tears, but optimal management of femoral‐side avulsion injuries remains challenging. The vascular femoral attachment site offers unique healing potential. However, conventional reconstruction requires extensive native tissue resection, sacrificing the remnant's proprioceptive function and biological healing capacity, making it suboptimal. The presented modified “suspender‐style” technique addresses this by combining native ligament repair via traction‐reduction with graft‐based reconstruction for mechanical support. This dual approach optimizes the healing microenvironment while maximizing proprioceptive preservation. Therefore, the surgical technique described in this article presents a viable surgical approach for anterior cruciate ligament reconstruction, applicable to Sherman type I injuries.

## Graphical abstract

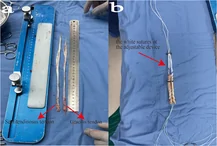

**VIDEO 1:** The anterolateral knee incision is used as the observation portal and the anteromedial knee incision as the working portal. First, with the knee flexed to 90°, the anterolateral portal is used for observation. Arthroscopic examination confirmed a femoral‐side avulsion of the anterior cruciate ligament (ACL). Second, with the knee flexed to 90°, the anteromedial portal is used as the working portal. A suture hook was used to pass a PDS‐II suture through the proximal one‐third of the ACL remnant, which was then employed to shuttle a #5 ETHIBOND suture (Ethicon, Raritan, NJ), creating a lasso‐loop around the avulsed stump. Third, with the knee flexed to 120°, the anterolateral portal is used for observation, the anteromedial portal serves as the working portal, and the femoral tunnel was established. A 6.0‐mm femoral aimer was introduced through the anteromedial portal and positioned at the center of the ACL footprint on the medial femoral condyle. A 2.0‐mm beaded guide pin was drilled, followed by overdrilling with a 4.5‐mm EndoButton (Smith & Nephew, Andover, MA) cannulated reamer. The total length of the femoral socket was measured as 36 mm using a depth gauge, after which an 8.0‐mm cannulated reamer was used to create the femoral tunnel to a depth of approximately 20 mm. The anteromedial portal is used as the working portal. A PDS‐II suture was shuttled through this tunnel via the beaded guide pin to serve as a passing suture. Fourth, for the tibial tunnel, with the knee at 90° of flexion, an ACL tibial aimer was placed through the anteromedial portal. It was oriented at approximately 30° relative to the tibial longitudinal axis and 50° relative to the tibial plateau, targeting the center of the native ACL tibial footprint. The tunnel was created by drilling a 2.0‐mm K‐wire followed by an 8.0‐mm cannulated reamer in an inferomedial‐to‐superolateral direction into the joint. Fifth, a suture retriever was then introduced through the tibial tunnel to pull the tails of the #5 ETHIBOND suture (Ethicon, Raritan, NJ) and the PDS‐II passing suture out of the joint. These were used to shuttle the #5 ETHIBOND suture (Ethicon, Raritan, NJ) and the passing suture of an adjustable suspensory fixation device. The PDS‐II suture was then pulled to deliver all suture tails out through the femoral tunnel and the lateral femoral cortex. Sixth, at the lateral skin surface overlying the distal femoral tunnel exit, the adjustable suspensory button was advanced through the femoral tunnel and flipped to engage securely against the lateral femoral cortex by pulling the device's passing suture. The white sutures of the device were alternately tensioned to advance the tendon graft into the femoral tunnel. Simultaneously, the #5 ETHIBOND suture (Ethicon, Raritan, NJ) tails were pulled to reduce the native ligament to its femoral footprint. Seventh, a knot pusher was used to tie the #5 ETHIBOND suture (Ethicon, Raritan, NJ) to the device's white sutures, creating the “suspender‐style” traction effect. After final graft tensioning, the knee was cycled through a full range of motion to assess for graft impingement. Finally, an arthroscopic evaluation was performed to confirm the satisfactory tension and anatomic position of both the reduced native ligament and the tendon graft. Video content can be viewed at https://doi.org/10.1002/atn2.70234.

Anterior cruciate ligament (ACL) rupture, one of the most frequent sports‐related knee injuries, predominantly occurs in athletic populations such as competitive athletes and young, physically active individuals.^1^, ^2^ After injury, it often manifests as significant anterior and rotational instability of the knee, restricted range of motion, and markedly impaired athletic function.^3^, ^4^ Moreover, it significantly elevates the risk of meniscal damage and articular cartilage injury, thereby increasing the long‐term incidence of secondary osteoarthritis. Therefore, active and effective intervention for ACL rupture is crucial for restoring knee function and preventing long‐term complications. In the clinical management of ACL rupture, remnant‐preserving anatomical reconstruction has become the first‐line treatment for midsubstance ACL tears. This procedure uses arthroscopic‐assisted autologous tendon grafting to reconstruct the ligament at its anatomical position, thereby restoring the biomechanical stability of the knee.^5^, ^6^ When an ACL rupture occurs at the femoral footprint, remnant‐preserving anatomical reconstruction typically requires the resection of a significant portion of the avulsed native ACL tissue. This is done to prevent notch impingement by the free end, preserving only a small portion of the remnant ligament tissue on the tibial side. Although this procedure achieves mechanical reconstruction, it largely sacrifices the native ligament's inherent proprioceptive nerve endings and biological healing potential.^7^ Proprioception is critical for joint position sense, kinesthesia, and neuromuscular control, serving as the foundation for achieving high‐level functional recovery.^8^, ^9^, ^10^ Therefore, for ACL ruptures occurring at the femoral footprint, direct resection of the native ligament may not represent the optimal therapeutic strategy.

In recent years, the goal of surgical treatment has evolved from purely mechanical reconstruction to restoring stability while striving to preserve and harness the biological function of native tissue. Several studies have reported the use of isolated anatomical repair for the treatment of femoral‐side avulsions of the ACL.^11^, ^12^, ^13^ Although this procedure can harness the biological function of native ligament tissue, it possesses an inherent and critical limitation: the repaired native ligament often shows insufficient mechanical strength, failing to withstand high‐intensity athletic loads. As a result, it does not meet the therapeutic demands of competitive athletes and patients with high activity levels, which restricts its clinical applicability. In pursuit of an optimal balance between mechanical strength, biological healing, and proprioceptive function, we describe the modified “suspender‐type” traction‐preservation and native ligament reconstruction technique (Video 1). This technique uses a “suspender‐type” traction‐reduction approach to accurately repair the femoral‐side avulsion of the native ACL, while concurrently incorporating a reconstruction component to establish robust mechanical support. It thereby creates a more favorable healing microenvironment for autograft integration while maximally preserving the proprioceptive function inherent to the native ligament.

This technical note focuses on the application of this technique for treating femoral‐side ACL avulsions. It aims to systematically elucidate its key procedural details, provide a superior surgical alternative for this specific injury pattern, and ultimately contribute to improved patient outcomes and successful return to sports.

## SURGICAL TECHNIQUE

**FIGURE 1:**
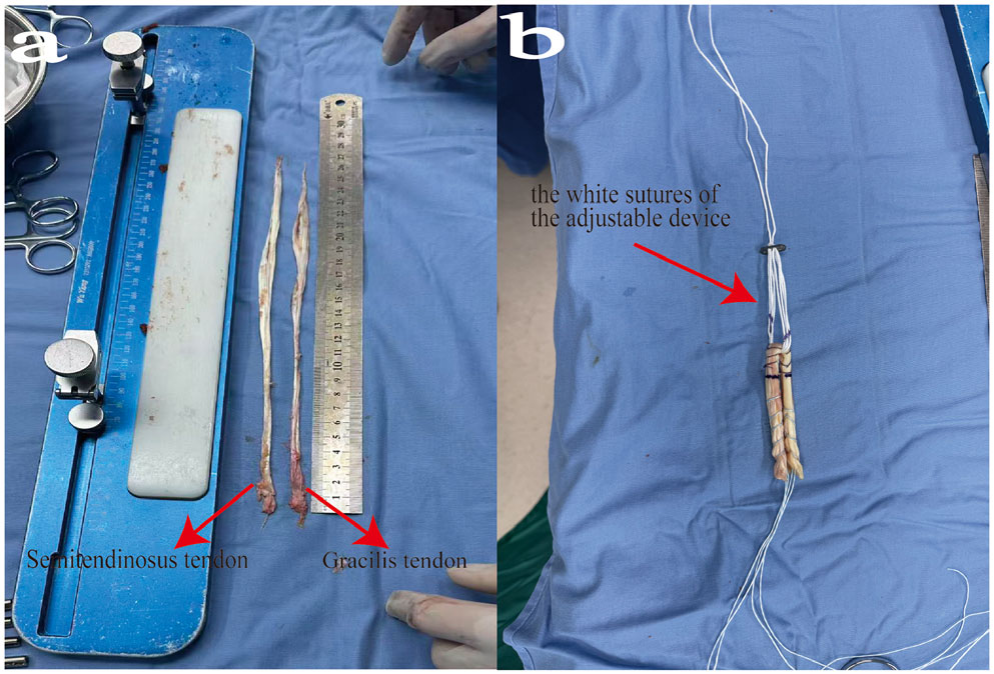
Graft preparation. (a) The harvested semitendinosus tendon (approximately 27 cm in length) and gracilis tendon (approximately 26 cm in length). (b) The prepared quadruple‐stranded tendon graft, looped onto an adjustable suspensory fixation device, measuring 8 cm in length and 8 mm in diameter.

**FIGURE 2:**
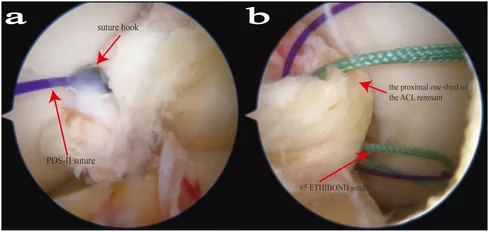
Suturing of the native ACL remnant. (a) A suture hook is used to pass a PDS‐II suture (violet) through the proximal one‐third of the ACL remnant. (b) The PDS‐II suture is then used to shuttle a #5 ETHIBOND suture (Ethicon, Raritan, NJ) (green), creating a lasso loop around the remnant for subsequent traction and reduction to the femoral footprint. (ACL, anterior cruciate ligament.)

**FIGURE 3:**
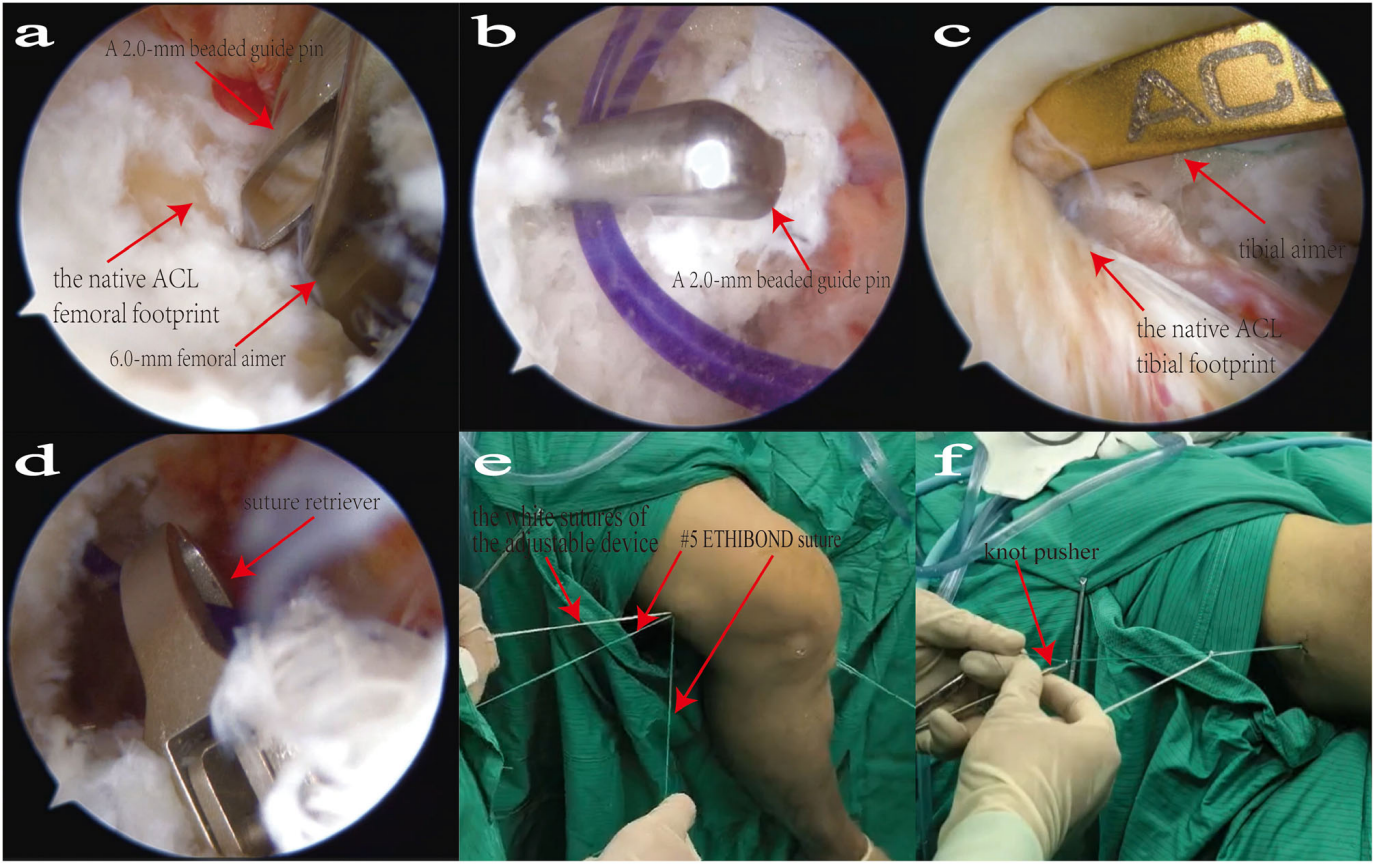
Surgical steps for tunnel creation and graft passage. (a) Through the anteromedial portal, a 6.0‐mm femoral aimer is positioned at the center of the ACL footprint on the medial femoral condyle. The femoral socket is then created sequentially using a 2.0‐mm beaded guide pin, a 4.5‐mm EndoButton (Smith & Nephew, Andover, MA) cannulated reamer, and an 8.0‐mm cannulated reamer. (b) A PDS‐II suture is shuttled through the femoral tunnel via the beaded guide pin to serve as a passing suture. (c) Through the anteromedial portal, a tibial aimer is positioned at the center of the native ACL tibial footprint. The tibial tunnel is created using a 2.0‐mm K‐wire followed by an 8.0‐mm cannulated reamer. (d) A suture retriever is introduced through the tibial tunnel to pull the tails of both the #5 ETHIBOND suture (Ethicon, Raritan, NJ) and the PDS‐II passing suture out of the joint, which will be used to shuttle the #5 ETHIBOND suture (Ethicon, Raritan, NJ) and the adjustable suspensory fixation device through the femoral tunnel. (e) The white sutures of the adjustable suspensory device are alternately tensioned to advance the tendon graft into the femoral tunnel. (f) A knot pusher is used to secure the #5 ETHIBOND suture (Ethicon, Raritan, NJ) to the white sutures of the adjustable device, achieving the “suspender‐style” traction‐reduction effect. (ACL, anterior cruciate ligament.)

**FIGURE 4:**
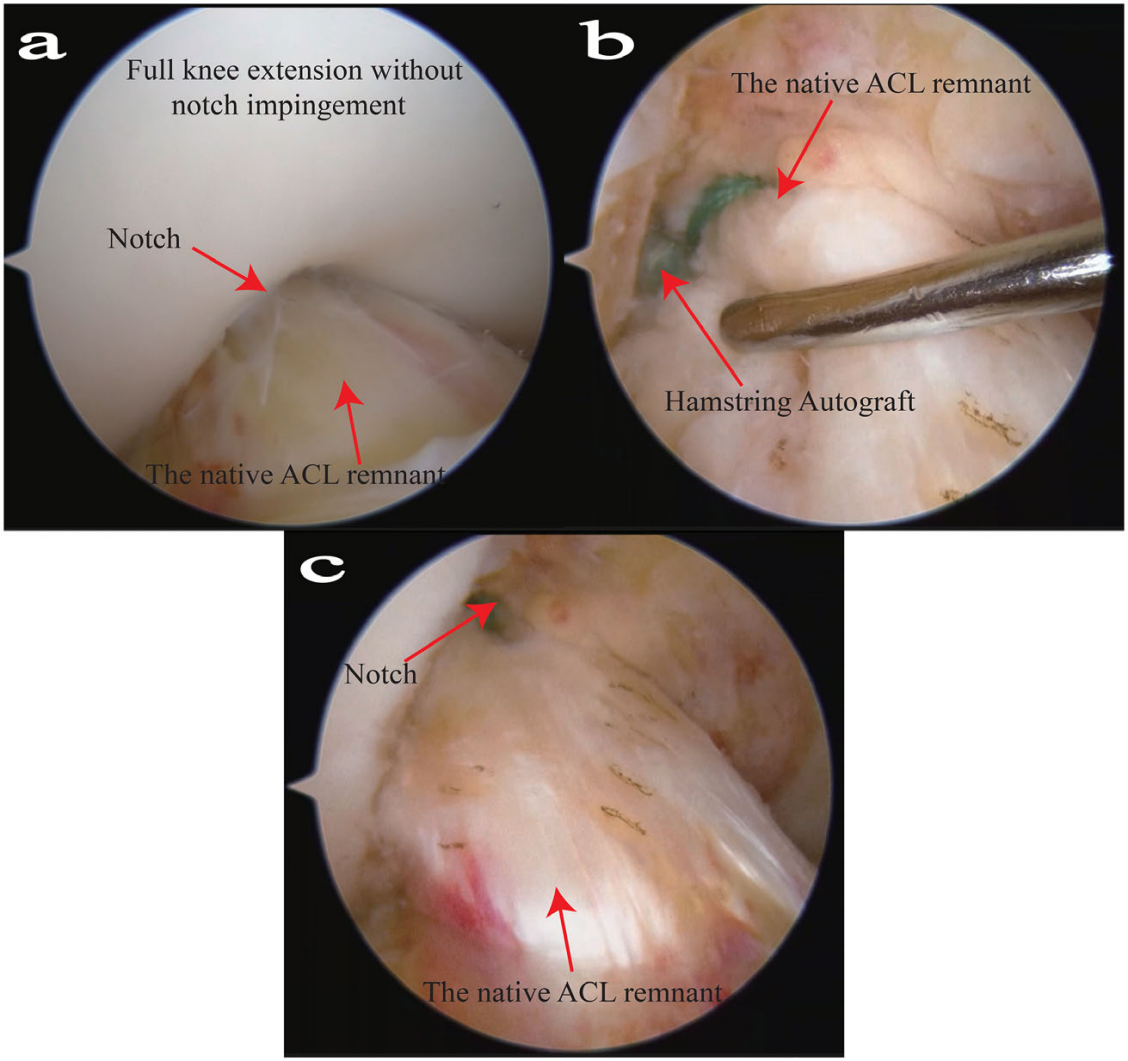
Final arthroscopic assessment. (a) With the knee in full extension, viewing from the anterolateral portal. The knee is cycled through a full range of motion to check for graft impingement within the intercondylar notch. (b) With the knee flexed to 90°, viewing from the anterolateral portal. Arthroscopic view showing the final tension and anatomic position of both the reduced native ligament and the tendon graft. (c) With the knee flexed to 90°, viewing from the anterolateral portal. Final arthroscopic overview on the completion of the modified “suspender‐style” traction‐preservation and native ligament reconstruction procedure. (ACL, anterior cruciate ligament.)

General anesthesia was administered with the patient in the supine position. After standard sterilization and draping, conventional anteromedial and anterolateral arthroscopic portals were established. Diagnostic arthroscopy was performed to confirm the ACL rupture and to evaluate for concomitant injuries, such as meniscal tears and cartilage lesions. First, a shaver was introduced to debride hypertrophic synovial tissue within the patellofemoral joint space and the anterior compartment of the knee. Hemostasis of the debrided synovial bed was achieved using a radiofrequency ablation electrode (BONSS, BDS313, Jiangsu, China). Second, any torn meniscus was repaired using a meniscal suture device (Smith & Nephew, FastFix 360°, Hertfordshire, United Kingdom). Third, a 3‐cm oblique skin incision was made centered on the intersection of the horizontal line at the level of the tibial tubercle apex and the vertical line along the medial border of the patella. After incising the skin and fascial layer, the semitendinosus and gracilis tendons were identified. Both tendons were bluntly dissected, looped with a tendon harvester, and harvested proximally at their musculotendinous junctions, obtaining the tendons with their periosteal attachments (Figure 1a). The harvested tendons were fashioned into an 8.0‐cm long, 8.0‐mm diameter graft for ACL reconstruction. Both ends of the graft were whipstitched with high‐strength suture over a length of 20 mm each (Figure 1b). Fourth, a suture hook was used to pass a PDS‐II suture (violet) through the proximal one‐third of the ACL remnant (Figure 2a). This PDS‐II suture was then used to shuttle a #5 ETHIBOND suture (Ethicon, Raritan, NJ) (green), which was subsequently employed to create a lasso‐loop configuration around the ACL remnant (Figure 2b). Fifth, the femoral tunnel was established. With the knee flexed at 120°, a 6.0‐mm femoral aimer was positioned at the center of the ACL footprint on the medial femoral condyle (Figure 3a). A 2.0‐mm beaded guide pin was drilled, followed by overdrilling with a 4.5‐mm EndoButton (Smith & Nephew, Andover, MA) cannulated reamer. The total length of the femoral socket was measured as 36 mm using a depth gauge. Subsequently, an 8.0‐mm cannulated reamer was used to create the femoral tunnel to a depth of approximately 20 mm. A PDS‐II suture was then shuttled through the anteromedial portal as a passing suture, traversing the femoral tunnel using the beaded guide pin (Figure 3b). Sixth, the tibial tunnel was established. With the knee flexed at 90°, an ACL tibial aimer was introduced through the anteromedial portal into the joint. The aimer was oriented at approximately 30° relative to the longitudinal axis of the tibia and approximately 50° relative to the tibial plateau. The tunnel entrance was positioned at the center of the native ACL tibial footprint (Figure 3c). A 2.0‐mm Kirschner (K)‐wire was drilled, followed by overdrilling with an 8.0‐mm cannulated reamer, to create the tibial tunnel in an inferomedial‐to‐superolateral direction into the joint cavity. Seventh, a suture retriever was introduced through the tibial tunnel to pull the trailing ends of both the #5 ETHIBOND suture (Ethicon, Raritan, NJ) (used to lasso the ligament remnant) and the PDS‐II passing suture out of the joint cavity and through the tibial tunnel (Figure 3d). The prepared tendon graft was then looped onto an adjustable suspensory fixation device (NATON, 4.4 × 12.2 mm, Beijing, China). Next, the #5 ETHIBOND suture (Ethicon, Raritan, NJ) and the passing suture of the adjustable device were threaded through the loop of the PDS‐II suture. The PDS‐II suture was then pulled to shuttle these suture tails out through the femoral tunnel and the lateral femoral cortex. Eighth, by pulling the passing suture of the adjustable device, the suspensory button was advanced through the femoral tunnel until its flip plate was deployed and seated flush against the outer cortex of the femoral tunnel opening. Subsequently, the white sutures of the adjustable device were alternately tensioned to advance the tendon graft into the femoral tunnel until the graft's marking suture reached the tunnel entrance (Figure 3e). Concurrently, the #5 ETHIBOND suture (Ethicon, Raritan, NJ) tails were pulled to reduce and approximate the native ligament remnant to its femoral footprint. A knot pusher was then used to tie the #5 ETHIBOND suture (Ethicon, Raritan, NJ) to the white sutures of the adjustable device, creating the “suspender‐style” traction effect (Figure 3f). Ninth, after final graft tensioning, the knee was cycled through the full range of motion to ensure the ACL graft was free from impingement or notch compromise (Figure 4a). Maintaining tension on the graft, a 9.0 × 25 mm bioabsorbable interference screw (Smith & Nephew, Hertfordshire, United Kingdom) was inserted into the tibial tunnel for tibial‐side fixation. Finally, the tension and anatomic position of both the native ligament and the tendon graft were assessed arthroscopically (Figure 4b). This completed the modified “suspender‐style” traction‐preservation and native ligament reconstruction procedure (Figure 4c).

## DISCUSSION

The management of femoral‐side ACL avulsions involves a critical balance between achieving mechanical stability, promoting biological healing, and preserving proprioceptive function. Unlike midsubstance tears, injuries at the femoral footprint benefit from the region's abundant vascularity, which provides a natural advantage for the biological healing of the ligament remnant. Furthermore, the native ligament is richly innervated with proprioceptive nerve endings—primarily Ruffini and Pacinian corpuscles—which are crucial for sensorimotor control of the knee and functional recovery.^7^, ^14^, ^15^ Although traditional remnant‐preserving anatomical reconstruction represents the first‐line treatment for midsubstance ACL tears, its application in this specific subtype necessitates the resection of a substantial portion of the avulsed native ligament. This inevitably leads to the loss of proprioceptive nerve endings and compromises the inherent biological healing potential, which may ultimately result in suboptimal long‐term functional recovery for patients. In contrast, isolated ACL repair offers the distinct advantage of achieving anatomical reduction of the ligament remnant to its femoral footprint. This approach maximizes the preservation of the remnant's vascular supply and proprioceptive neural pathways.^16^, ^17^ Basic studies have confirmed that the preserved ligament remnant is rich in fibroblasts and vascular endothelial cells, which can regulate the local inflammatory microenvironment through paracrine signaling, thereby providing progenitor cells and nutritional support for ligament healing. Moreover, the intact preservation of proprioceptive receptors—such as Ruffini and Pacinian corpuscles—within the remnant serves as the critical anatomical foundation for maintaining precise neuromuscular control of the knee postoperatively.^18^, ^19^, ^20^ However, extensive clinical experience has shown that the mechanical strength of the isolated repair is sufficient only for activities of daily living and cannot withstand the loads associated with high‐intensity sports. Consequently, this procedure carries a relatively high risk of re‐rupture, particularly for athletes and other young, active individuals.^21^, ^22^, ^23^ Based on this rationale, the modified “suspender‐style” traction‐preservation and native ligament reconstruction technique proposed in this study embodies the core philosophy of “reconstruction and repair,” achieving their effective integration and thereby providing an optimized solution for the treatment of femoral‐side ACL avulsions.

The modified technique presented in this study integrates the advantages of mechanical stability, biological healing, and preserved proprioceptive function (Table 1). On one hand, the “suspender‐style” traction‐reduction strategy maximizes the retention of the native ACL tissue's biological value. During the procedure, a #5 ETHIBOND suture (Ethicon, Raritan, NJ) is used to create a lasso‐loop around the proximal one‐third of the ACL remnant. This suture is then employed via the “suspender‐style” method to achieve anatomical reduction and fixation of the remnant to its femoral footprint. This approach avoids the extensive resection of the native ligament stump required in traditional reconstruction, fully leveraging the rich vascularity of the femoral footprint to create a favorable microenvironment for native ligament healing. Concurrently, it preserves the proprioceptive nerve endings within the native ligament, which are crucial for restoring joint position sense, kinesthesia, and neuromuscular control. This preservation serves as the foundation for high‐level functional recovery and successful return to sports. On the other hand, the concomitant autologous tendon reconstruction establishes robust mechanical support. In this study, grafts prepared from the semitendinosus and gracilis tendons were used, which offer excellent biocompatibility and mechanical strength. This effectively compensates for the insufficient mechanical strength inherent in isolated repair techniques. Furthermore, the standardization of the key procedural steps is crucial for ensuring the efficacy of this technique (Table 2).

**TABLE 1:** Advantages and Disadvantages

Advantages	Disadvantages
Integrates biological repair (preservation) with mechanical reconstruction	Primarily suitable for acute femoral‐side avulsions/proximal tears with viable remnants; less advantageous for chronic injuries or poor‐quality remnants
Retains the native ligament's proprioceptive nerve endings and vascular supply, enhancing healing potential and sensorimotor recovery	Absence of long‐term (e.g., 1 and 3 yrs) follow‐up studies to validate sustained efficacy
Provides robust mechanical support via autologous tendon graft, addressing the weakness of isolated repair	Current study has a small sample; requires larger, multicenter trials for broader validation
Accurate tunnel placement and ligament reduction help restore native ACL biomechanics and reduce instability risks	No direct comparison with conventional reconstruction or isolated repair in this study, limiting comparative efficacy conclusions
Favorable for active populations: particularly beneficial for young, active individuals and athletes aiming for high‐level functional return	Success depends on precise, gentle handling of the native remnant and exact tunnel positioning, requiring a learning curve
	The preservation of the native ligament remnant on the tibial side may lead to inaccurate tibial tunnel positioning by less experienced surgeons

ACL, anterior cruciate ligament.

**TABLE 2:** Pearls and Pitfalls

Pearls	Pitfalls
Suitable for Sherman type I avulsions, requiring good quality and healing potential of the ACL remnant	Not recommended for chronic ACL tears or Sherman type II and III injuries
Suture ligation of the ACL remnant should be placed at least at the proximal one‐third, ensuring even traction force for reduction	Insufficient suture purchase may lead to remnant tear or inadequate reduction and fixation
Femoral tunnel: knee flexed at 120°, positioned at the center of the ACL femoral footprint on the lateral femoral condyle Tibial tunnel: knee flexed at 90°, positioned at the center of the native ACL tibial footprint	The presence of an intact tibial ACL remnant may obscure visualization, leading to inaccurate tibial tunnel placement by less experienced surgeons
Diameter of the autologous hamstring tendon graft should ideally be between 7 and 9 mm	A diameter <7 mm may cause graft‐tunnel micromotion (windshield‐wiper effect) and tunnel widening. A diameter >9 mm may risk notch impingement or tunnel fracture
While pulling the graft into the femoral tunnel, simultaneously tension the ETHIBOND sutures (Ethicon, Raritan, NJ) to reduce the native ligament remnant to its femoral footprint	Insufficient traction or insecure suture fixation may prevent the remnant from achieving close apposition to the bone, compromising healing
Use a knot pusher to securely tie the ETHIBOND sutures (Ethicon, Raritan, NJ) to the traction sutures of the adjustable‐loop device, creating a stable “suspender‐like” suspensory fixation	Loose knots or suture slippage can lead to remnant retraction, loss of the repair effect, and potential notch impingement
The interference screw is inserted into the tibial tunnel with the graft under appropriate tension	Insufficient initial graft tension may result in postoperative laxity
The knee must be taken through a full range of motion under arthroscopy to confirm the absence of notch impingement and to assess the tension and position of both the native remnant and the graft	Failure to dynamically check for impingement may lead to graft abrasion against the intercondylar notch, causing pain, graft laxity, or failure

ACL, anterior cruciate ligament.

This study has several limitations. First, the technique is primarily indicated for patients with femoral‐side ACL avulsions or proximal midsubstance tears, provided the remnant tissue is of sufficient quality and healing potential. Its advantages diminish considerably in cases of chronic injuries or when the remnant is severely absorbed or of poor quality, where conventional reconstruction remains a more reliable option. Second, this study lacks long‐term follow‐up data. Further investigation with clinical outcomes at 1, 3, and more years postoperatively is necessary to validate the technique's long‐term efficacy. The sample size also requires expansion; future multicenter, large‐scale clinical studies are needed to better establish its generalizability. Finally, the absence of a control group (e.g., patients treated with conventional reconstruction or isolated repair) limits direct comparative analysis. Subsequent controlled studies are warranted to clarify the differences between this technique and existing procedures regarding clinical outcomes, mechanical stability, and long‐term prognosis.

The modified “suspender‐type” traction‐preservation and native ligament reconstruction technique presented in this study not only preserves the proprioceptive function and vascular advantages of the native ACL but also provides adequate mechanical strength via autologous tendon grafting, thereby establishing favorable conditions for postoperative functional recovery and return to sports. This standardized technique shows good potential for clinical adoption, offering a surgical alternative for patients with ACL Sherman type I injuries.

## DISCLOSURES

The authors (H.Y., X.Q.) declare that they have no known competing financial interests or personal relationships that could have appeared to influence the work reported in this article.

## FUNDING

This work was sponsored by Self‐financed Scientific Research Project of the Health Commission of Guangxi Zhuang Autonomous Region No. Z‐B20221442.
